# Expression of Concern on “Influence of gastrectomy on cortical and cancellous bones in rats”

**DOI:** 10.1155/2019/4312310

**Published:** 2019-07-07

**Authors:** Gastroenterology Research and Practice

*Gastroenterology Research and Practice* would like to express our concern with the article titled “Influence of gastrectomy on cortical and cancellous bones in rats”, published in 2013 [1]. It was brought to our attention that a later article by the same authors published in *Journal of Nutritional Science and Vitaminology* in 2014 [2] used some of the same animals as in the *Gastroenterology Research and Practice* article, but the second article had an additional arm of rats given vitamin K and did not cite the first article. The authors did not mention in the *Gastroenterology Research and Practice* article that they had also used an additional arm of rats given vitamin K. In addition to the partial redundant publication, readers should be aware of the following concerns and discrepancies:

1. In Figure 1 of *Journal of Nutritional Science and Vitaminology*, the gastrectomy data for all six variables are discrepant with those for the same variables in Figure 3 of *Gastroenterology Research and Practice*
2. In Figure 1 of *Journal of Nutritional Science and Vitaminology*, the control data for work to failure in the femoral diaphysis are discrepant with those for the same variable in Figure 3 of *Gastroenterology Research and Practice*
3. In the legends to Figures 1, 2, and 3 in *Gastroenterology Research and Practice*, the superscript letters denoting statistical significance are stated to be related to ‘versus gastrectomy' though they are above the gastrectomy data. This should probably have read ‘versus control';
4. In *Journal of Nutritional Science and Vitaminology*, control and gastrectomy groups had vehicle administered by oral gavage, but in *Gastroenterology Research and Practice* there is no mention of vehicle administration in either group
5. No funding statement was provided
6. The study was stated to have been conducted at Hamri Co (Ibaraki, Japan), yet none of the authors had affiliations there and neither did any of the acknowledged contributors
7. Co-author Yoshihiro Sato, since deceased, previously admitted he was given gift authorship on other articles by Jun Iwamoto

We asked the first author, Jun Iwamoto, to respond to these concerns and provide the raw data, but he did not explain the discrepancies or provide the data. We asked the Dean of Keio University School of Medicine and the Provost of Keio University to investigate. We were informed by a contact from the Research Administration at Keio University School of Medicine that an investigation was complete and we would be contacted by Dr. Iwamoto, but we were not told the outcome of the investigation and our specific concerns were not addressed.

Dr. Iwamoto provided the raw data for both articles (sending one set of spreadsheets with data comparing controls versus gastrectomy, and other set of spreadsheets comparing controls versus gastrectomy versus gastrectomy plus vitamin K, all available as supplementary files (available here)), but he did not explain which data were associated with which of the results in the articles. The control results and data are the same in the two articles and spreadsheets. However, the gastrectomy results differ between the spreadsheets, because not all the same animals were reported in each data set: for the *Gastroenterology Research and Practice* article, the animals are coded 201; 202; 203; 204; 205; 206; 207; 208; 209; 210, whereas for the *Journal of Nutritional Science and Vitaminology* article they are coded 201; 203; 204; 205; 206; 208; 209; 210; 211; 212 (missing 202 and 207, and instead including 211 and 212). This difference in the animals used between the two spreadsheets has not been explained or justified, but (assuming that the controls versus gastrectomy spreadsheet corresponds to the *Gastroenterology Research and Practice* article and the controls versus gastrectomy versus gastrectomy plus vitamin K spreadsheet corresponds to the *Journal of Nutritional Science and Vitaminology* article) it is a potential reason for the discrepancies noted above between the results in the two articles.

However, the raw data for the gastrectomy group in the controls versus gastrectomy spreadsheet provided by the author does not match the results in the *Gastroenterology Research and Practice* article. For example, the means and standard deviations for the initial and final body weights in the raw data in the spreadsheet provided by the author – 387 ± 16 and 402 ± 37 – do not match those reported in the article – 383 ± 14 and 404 ± 39. However, the gastrectomy body weight data in the controls versus gastrectomy versus gastrectomy plus vitamin K spreadsheet do match the results in *Gastroenterology Research and Practice*. In that case, there should have been no discrepancies between the results reported in the two articles. It is unclear to what extent these discrepancies affect the results of this article.

## Supplementary Materials

Supplementary Materials The files include the data of the two studies.
